# A Modular Toolset for Recombination Transgenesis and Neurogenetic Analysis of *Drosophila*

**DOI:** 10.1371/journal.pone.0042102

**Published:** 2012-07-25

**Authors:** Ji-Wu Wang, Erin S. Beck, Brian D. McCabe

**Affiliations:** Center for Motor Neuron Biology and Disease, Department of Pathology and Cell Biology, Department of Neuroscience, Columbia University, New York, New York, United States of America; University of Houston, United States of America

## Abstract

Transgenic *Drosophila* have contributed extensively to our understanding of nervous system development, physiology and behavior in addition to being valuable models of human neurological disease. Here, we have generated a novel series of modular transgenic vectors designed to optimize and accelerate the production and analysis of transgenes in *Drosophila*. We constructed a novel vector backbone, pBID, that allows both phiC31 targeted transgene integration and incorporates insulator sequences to ensure specific and uniform transgene expression. Upon this framework, we have built a series of constructs that are either backwards compatible with existing restriction enzyme based vectors or utilize Gateway recombination technology for high-throughput cloning. These vectors allow for endogenous promoter or Gal4 targeted expression of transgenic proteins with or without fluorescent protein or epitope tags. In addition, we have generated constructs that facilitate transgenic splice isoform specific RNA inhibition of gene expression. We demonstrate the utility of these constructs to analyze proteins involved in nervous system development, physiology and neurodegenerative disease. We expect that these reagents will facilitate the proficiency and sophistication of *Drosophila* genetic analysis in both the nervous system and other tissues.

## Introduction

The ability to express an exogenous gene in a transgenic animal with control over the timing, level and pattern of expression is essential for many types of experimental analysis. The sophistication of *Drosophila* genetics combined with the advent of transformation with transposable elements vectors [1] followed by subsequent innovations such as the Gal4/UAS system [2] have made *Drosophila* a powerful model system in which to address a multitude of biological problems including the development and function of the nervous system [3]. More recently *Drosophila* transgenic technology has been further improved by the ability to reproducibly target transgenes to specific genomic loci using ϕC31 phage integrase based DNA recombination [4]. ΦC31 integrase catalyzes the recombination between a phage attachment site (*attP*) and a bacterial attachment site (*attB*) [5]. For *Drosophila* transgenesis, an *attP* site is introduced into the genome using conventional transposon techniques [4]. Injected plasmids containing an *attB* site can then integrate at this ‘landing’ or ‘docking’ site when ΦC31 integrase is provided from either a co-injected mRNA [4] or a transgenic source [6]. The integration event is both highly efficient and unidirectional with integrated transgenes remaining stable in the presence of integrase [7].

One drawback of the φC31 transgenesis technique is that the level of expression of integrated transgenes can differ depending upon the genomic location of the landing site. This can be somewhat mitigated by screening for landing sites that allow high levels of expression [6], [8], [9] but considerable variation still exists in tissue specific expression levels from integration sites situated at different genomic locations [10] presumably due to local chromatin influences. To counteract these position effects, insulator elements [11], which when surrounding a transgene can protect its expression from positive and negative chromatin effects, have been employed in both P-element [12], [13] and ϕC31 based transgenic vectors [9]. However the interaction of these insulator elements with the Hsp70 based promoter sequences used in these vectors can produce unwanted gene expression ‘leak’ in some tissues such as salivary glands from Upstream Activation Sequence (UAS) containing transgenes in animals that are not producing Gal4 [9], [14]. This limitation not only limits the usefulness of these vectors but also makes it difficult to generate ‘toxic’ transgenes where unwanted expression could affect animal viability.

Here we have constructed a new series of *Drosophila* ϕC31 transgenesis compatible vectors designed to allow specific and uniform transgene expression from different *attP* integration sites. Modifying a minimal *attB* containing vector that carries a mini-*white* gene [6], we have added gypsy insulator sequences [15] to surround either a multiple cloning site that is backwards compatible with the popular P-element vector pUAST [2] or a high-throughput Gateway recombination cloning site [16]. From these starting backbones, which we call pBID (att**B**, **I**nsulated, ***D*** *rosophila*), we have built new GAL4 inducible vectors where we have replaced Hsp70 promoter sequences with a *Drosophila* Synthetic Core Promoter (DSCP) [17] to avoid unwanted leaky expression and added additional UAS binding sites to overcome the lower activity of this promoter. We show these vectors allow equivalent expression from commonly used *attP* landing sites and facilitate transgenic toxin generation. Building upon these constructs, we also generated novel UAS vectors that allow simple N or C-terminal fusion of transgenes to bright fast folding variants of Yellow or Red Fluorescent Proteins for *in vivo* imaging or protein epitopes for immunohistochemical or biochemical experiments. We find no evidence of unwanted expression from transgenes generated with these vectors in the absence of Gal4. Finally, we have used the pBID backbone to generate a vector that allows the rapid construction of UAS driven inverted hairpin sequences and show that these can be used for transgenic RNA inhibition (RNAi) of alternative splice isoforms of endogenous genes as a complement to existing whole genome libraries [18], [19]. We predict that these new reagents will expand the facility and usefulness of ϕC31 transgenesis for both neurogenetic and other applications in *Drosophila*.

## Results

**Figure 1:**
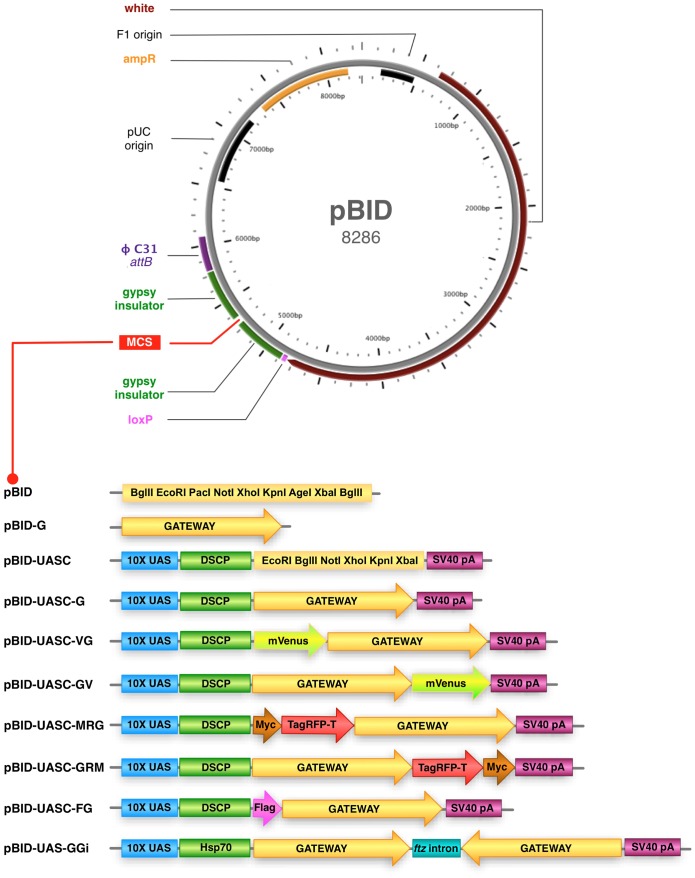
Overview of pBID vectors. **Above:** Map of the pBID vector (to scale). pBID includes a mini-*white* gene, a ϕC31 integrase compatible *attB* sequence (ϕC31 attB), a *loxP* site and an ampicillin resistance (ampR) gene. The multiple cloning site (MCS) is surrounded by gypsy insulator sequences to protect against genomic position effects. **Below:** Schematic of pBID vectors (not to scale). pBID has a restriction enzyme cloning site while pBID-G has a Gateway (G) cloning cassette. These vectors are suitable for cloning genomic fragments among other purposes. pBID-UASC has 10 copies of the Upstream Activation Sequence (10xUAS) Gal4 binding sequence, a *Drosophila* Synthetic Core Promoter (DSCP) basal promoter, a restriction enzyme MCS and an polyadenylation signal signal (SV40 pA). pBID-UASC-G has a Gateway cloning cassette. These vectors are suitable for Gal4 regulated transgene production. pBID-UASC-VG allows readthrough from mVenus (V) for fusion in frame to the N-terminus of genes introduced by Gateway cloning, while pBID-UASC-VG allows mVenus to be fused to the C-terminus of genes cloned by Gateway. pBID-UASC-MRG allows a Myc epitope and TagRFP-T (tRFP) to be fused to the N-terminus of genes introduced by Gateway cloning while pBID-UASC-GRM allows fusion to the C-terminus of Gateway cloned genes. pBID-UASC-FG allows a Flag epitope to be fused to the N-terminus of genes introduced by Gateway cloning. These vectors are suitable for Gal4 regulated fusion transgene expression. pBID-UAS-GGi has a Hsp70 basal promoter and two inverted Gateway cassettes separated by an intron from the *ftz* gene. This vector is suitable for the production of RNA hairpins for targeted RNAi inhibition of gene expression.

*Drosophila* transgenes, including constructs introduced by ϕC31 transgenesis [4], can have different levels of expression depending upon the genomic location of the landing site [9] and these differences persist even at sites of relatively high expression such as commonly used *attP18* on the site on the X chromosome, *attP40* site on chromosome 2 and the *attP2* site on chromosome 3 [9], [10]. To construct a vector that allows uniform transgene expression between landing sites, we wished to surround inserted genes with insulator elements to protect against local chromatin influences [11]. The gypsy insulator has been demonstrated to effectively inhibit both chromosome position effects [20] and modification by cis-regulatory elements [21]. We amplified the gypsy insulator sequence and subcloned a copy flanking either side of a multiple cloning site (MCS) into a backbone derived from pattB [6] to generate pBID (att**B**, **I**nsulated, ***D*** *rosophila*) (Figure 1A). The final pBID vector included an attB fragment that allows integration into the genome at *attP* landing sites, a mini-*white* gene to allow selection of transformed animals and an ampicillin resistance gene for plasmid selection. It also includes a loxP site which facilitates elimination of transgene markers via Cre recombinase-mediated excision when used in combination with *ZH-attP* landing sites [6]. The multiple cloning site of pBID was designed to contain similar restriction sites to the popular P-element vector pUAST [2] facilitating easy transfer of inserts from this vector to pBID in order to exploit the advantages of ϕC31 targeted transgenesis with some additional sites, such as the rare cutter PacI, introduced to further facilitate restriction enzyme based cloning (Figure 1B).

We first assayed if the pBID vector was capable of ϕC31 transgenesis and determined if transgenes were functional by using it to clone a genomic fragment encompassing the *caz* gene [22], the *Drosophila* ortholog of the ALS-associated gene FUS/TLS [23]. We injected this construct into strains carrying the *attP2* landing site and successfully identified integrants using *white* selection. We then determined if the *caz* genomic insert in pBID was functional by using these transgenes to rescue *caz* mutants, the majority of which die before adult eclosion [22]. We found the pBID *caz* genomic transgene could fully rescue *caz* mutant adult eclosion to control levels (Table 1), confirming that inserts in pBID were functional. Recombination based subcloning techniques, such as Gateway cloning, allow high throughput simultaneous transfer of inserts into multiple vectors and can facilitate the cloning of large inserts or sequences that are otherwise refractory to conventional cloning techniques [16]. To adapt pBID as a ‘destination’ vector compatible with Gateway cloning, we introduced a Gateway cassette into pBID to generate pBID-G. We confirmed, as expected, that pBID-G, like pBID, was also capable of producing functional transgenic inserts (data not shown). Therefore constructs introduced by both Gateway or conventional cloning techniques are functional in pBID.

**Table 1:** *caz* mutants are rescued by transgenic genomic *caz* generated with pBID.

Genotype	Eclosion (% of pupae)
Control	94+/−2.9
*caz^1^*/Y	4+/−0.9
*caz^1^*/Y;;genomic *caz*	89+/−2.1

A genomic fragment including the *caz* coding region and promoter [22] cloned into pBID and integrated into the *attP2* landing site on chromosome III can restore normal adult eclosion frequency when introduced into *caz* mutants.

### pBID-UASC Constructs have Equivalent Expression Levels at Different *Attp* Sites

We next designed UAS versions of the pBID vector. As insulator elements have been associated with GAL4 independent expression from UAS vectors that employ *hsp70* based promoters [9], [14] we decided to construct our vectors using a *Drosophila* Synthetic Core Promoter as the basal promoter [17]. One disadvantage of this promoter is that it produces lower transgene expression than the hsp70 basal promoter [10]. Therefore in order to increase transgene expression levels, we added 5 additional UAS binding sites more than are employed in the pUAST vector [2] for a total of 10 GAL4 binding sites. We called this vector pBID-UASC (**UAS** **C**ore promoter). We also introduced a Gateway cloning cassette into this vector to generate pBID-UASC-G.

**Figure 2:**
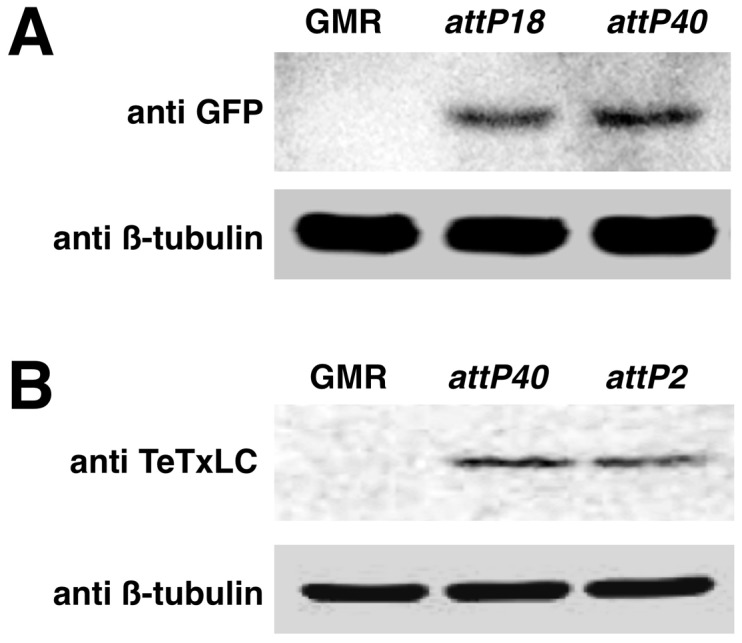
Expression of pBID transgenes is similar at different *attP* sites. **A.** Expression of destabilized GFP protein in adult heads from pBID-UASC inserts on the X chromosome (*attP18*) and chromosome II (*attP40*) driven by GMR-Gal4. The expression level is similar. The loading control is anti β-tubulin. **B.** Expression of Tetanus Toxin Light Chain (TeTxLC) in adult heads from pBID-UASC inserts on chromosome II (*attP40*) and chromosome III (*attP2*) driven by GMR-Gal4. Expression level is appears equivalent. Loading control is anti β-tubulin.

We first examined the expression level of transgenes inserted at different *attP* landing sites using these vectors. To do this we used pBID-UASC-G to generate transgenes with a destabilized form of EGFP (dsEGFP) which has a protein half-life of approximately two hours providing a sensitive measure of transgene expression [24]. We then generated transgenic insertions of UASC dsEGFP at *attP18* (X) and *attP40* (2) landing sites. When we examined by western blot analysis the level of dsEGFP protein expressed from either line when driven in eye tissues with GMR-Gal4, we found similar levels of dsEGFP protein expression were produced by either insert (Figure 2A). We also cloned tetanus toxin light chain (TeTxLC) [25], a toxin widely used for *Drosophila* behavioral studies [26], into pBID-UASC-G and generated inserts in *attP40* (2) or *attP2* (3) landing sites. Expression of TeTxLC from these lines completely inhibited neurotransmitter release when expressed in motor neurons similar to existing p-element based lines (data not shown). Similar to dsEGFP, when driven with GMR-Gal4, we also did not observe any difference in the level TeTxLC protein expressed from inserts at either *attP40* or *attP2* (Figure 2B). These results established that the insulator elements in pBID-UASC vectors allow for similar expression from different commonly used *attP* landing sites.

### pBID Vectors to Facilitate Transgene Fusion to Fluorescent and Epitope Protein Tags

**Figure 3:**
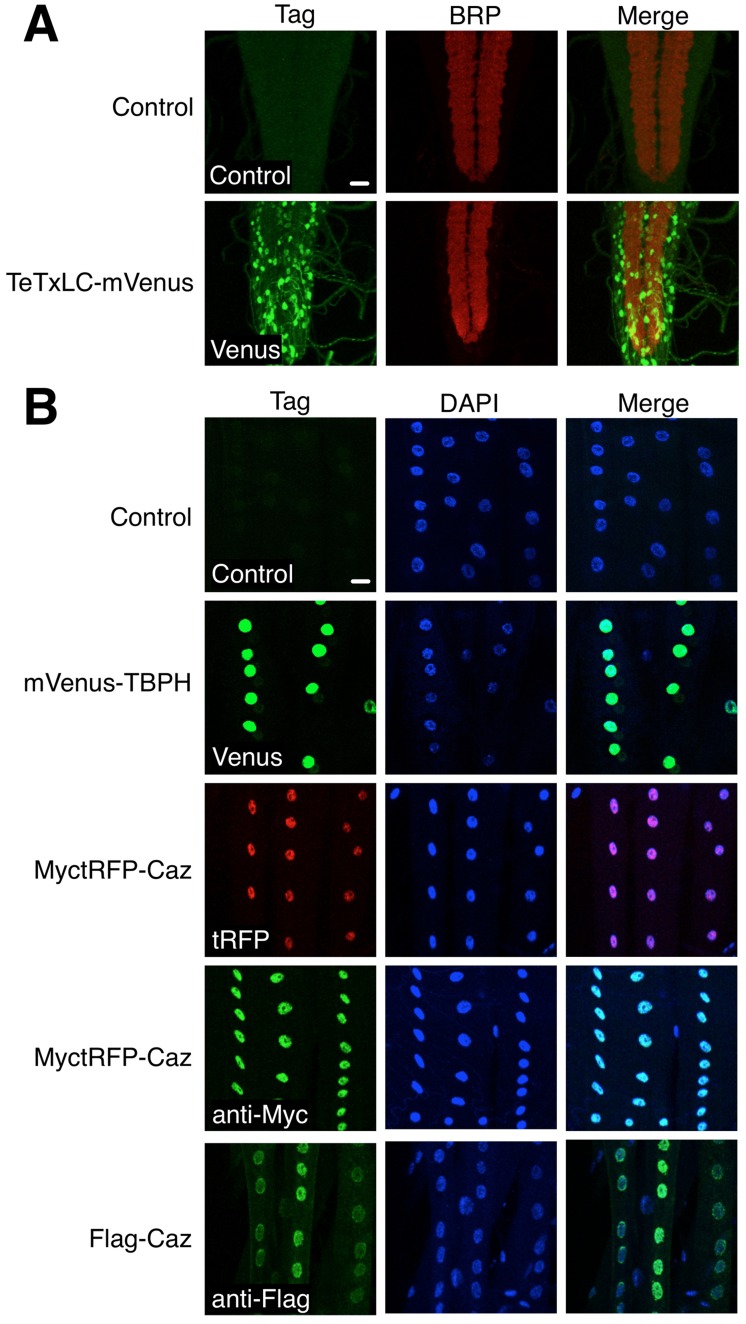
pBID vectors allow fusion of transgenes to fluorescent or epitope tags. **A.** mVenus (green) fused to the C-terminus of TeTxLC labels motor neurons in the larval ventral nerve cord when crossed with OK6-Gal4. The synaptic neuropile is labeled with anti-Brp (red). No signal is detectible in control animals. Scale Bar  = 20 µm. **B.** mVenus (green) fused to the N-terminus of TBPH or MyctRFP (red) fused to the N-terminus of Caz are found in muscle nuclei when expressed with G14-Gal4. MyctRFP-Caz is also detectible with anti-Myc (green). Similarly Flag fused to the N-terminus of Caz is also detectible with anti-Flag (green). Muscle nuclei are labeled with DAPI (blue). Scale Bar  = 20 µm.

We next generated pBID-UASC-G vectors designed to enable convenient fusion of transgenes to fluorescent proteins or epitope tags. We introduced the fast folding Yellow Fluorescent Protein (YFP) variant mVenus [27] in frame with the Gateway cassette to allow fusion of mVenus at the N-terminus (pBID-UASC-VG) or C-terminus (pBID-UASC-GV) of Gateway cloned transgenes. We confirmed these vectors produced functional transgenic fusions by generating a transgenic C-terminal fusion of mVenus to TeTxLC. Expression of this transgene with OK6-Gal4 showed strong mVenus fluorescence as expected in motor neurons (Figure 3A). Similarly, when mVenus was fused to the N-terminus of TBPH, the *Drosophila* homolog of the Amyotrophic Lateral Sclerosis associated protein TDP-43, we observed strong fluorescence as predicted in the nuclei of muscles when expressed with the muscle driver G14-Gal4 [28] (Figure 3B). We also generated vectors designed to allow N (pBID-UASC-MRG) or C terminal (pBID-UASC-GRM) fusion with TagRFP-T (tRFP), a bright monomeric red fluorescent protein [29], [30] to which we added a myc epitope tag to allow easy for immunohistochemical detection [31]. To confirm this fusion vector was effective, we generated transgenic *Drosophila* where MyctRFP was fused to the N-terminus of Caz. Expression of this fusion protein in muscles revealed bright red fluorescence in muscle nuclei as expected (Figure 3B). Using an anti-Myc antibody, we also could detect the MyctRFP-Caz fusion protein in the nucleus (Figure 3B). Finally, we generated a vector that allows easy transgene fusion to a Flag epitope (pBID-UASC-FG) [32] to facilitate biochemical purification in addition to immunohistochemical detection. When we generated transgenes where Caz was fused to Flag, we could also detect the protein in the muscle nucleus with anti-Flag antibody (Figure 3B) and purify the protein by immunoprecipitation (data not shown, [22]). Therefore, our vectors were effective at generating transgenic *Drosophila* fusion proteins that allow either fluorescent or epitope detection of protein localization.

### pBID UASC Vectors Lack Detectible Non-specific Transgene Expression

**Figure 4:**
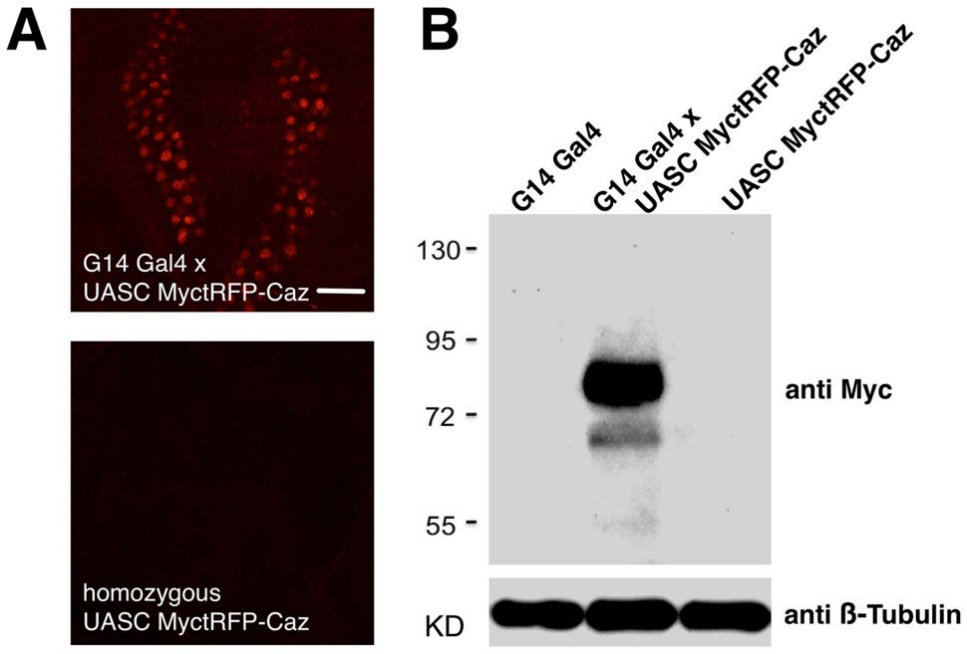
UAS pBID transgenes are not expressed in the absence of Gal4. **A.** Fluorescence from MyctRFP-Caz in *attP40* is easily observed in the salivary gland nuclei of larva when crossed to G14-Gal4. In contrast, no fluorescence is observable in homozygous inserts of MyctRFP-Caz in the absence of a Gal4 driver. Scale Bar  = 100 µm. **B.** MyctRFP-Caz protein expression is easily detectible with anti-Myc antibody in extracts from larva that carry G14-Gal4. In contrast, no expression is detectible in homozygous MyctRFP-Caz larva or G14 Gal4 control larva. Additional bands are due to overexposure of the blot. Loading control is anti β-tubulin.

A problematic issue identified with some previous UAS insulated vectors has been expression of transgenes in the absence of GAL4 [9], [14] impeding the usefulness of these vectors. To determine if our vectors based on the DSCP basal promoter also had this issue, we examined the expression of UASC MyctRFP-Caz with and without Gal4. In the presence of G14-Gal4, MyctRFP-Caz was detected in both muscles and the salivary glands, a tissue where many GAL4 lines are expressed [33] and where GAL4 independent leak of insulated UAS transgenes occurs (Figure 4A) [14]. In contrast, in the absence of GAL4, we could detect no fluorescence even in animals with 2 copies of UASC-MyctRFP-Caz in *attP2*. We observed similar results with fluorescent fusion transgenes inserted at *attP40* or *attP18* (data not shown). To examine if leaky expression could be occurring below our detection threshold by light microscopy, we homogenized and generated protein extracts from whole larvae with UASC-MyctRFP-Caz transgenes in *attP2* with or without G14 Gal4 (Figure 4B). We could detect abundant MyctRFP-Caz at the expected molecular weight by western blot using anti-Myc antibody in protein extracts from larva with G14 Gal4. In contrast, we could not detect any protein from extracts of larva with in control G14 GAL4 alone or 2 copies of UASC-MyctRFP-Caz alone. Therefore we conclude that, within the limits of our detection sensitivity, pBID-UASC generated transgenes do not aberrantly express transgenes in the absence of Gal4.

### pBID-UAS-GGi Allows Isoform Specific RNAi Inhibition of Gene Expression

Transgenic RNAi inhibition is a powerful technique for functional analysis of *Drosophila* genes which has been greatly facilitated by the generation of genome-wide libraries [18], [34]. However, many *Drosophila* genes have alternative splice isoforms with unique expression or functions which are not currently addressable using these libraries [35]. We therefore constructed a pBID vector designed to allow easy construction of RNA hairpins to target individual splice isoforms of *Drosophila* genes. To do this we constructed pBID-UAS-GGi which has two Gateway cassettes oriented in opposite directions separated by a *ftz* intron [36]. By this design, two inverted sequences are introduced simultaneously by the Gateway cloning reaction allowing the expression of an RNA hairpin that will target the cloned sequence.

**Figure 5:**
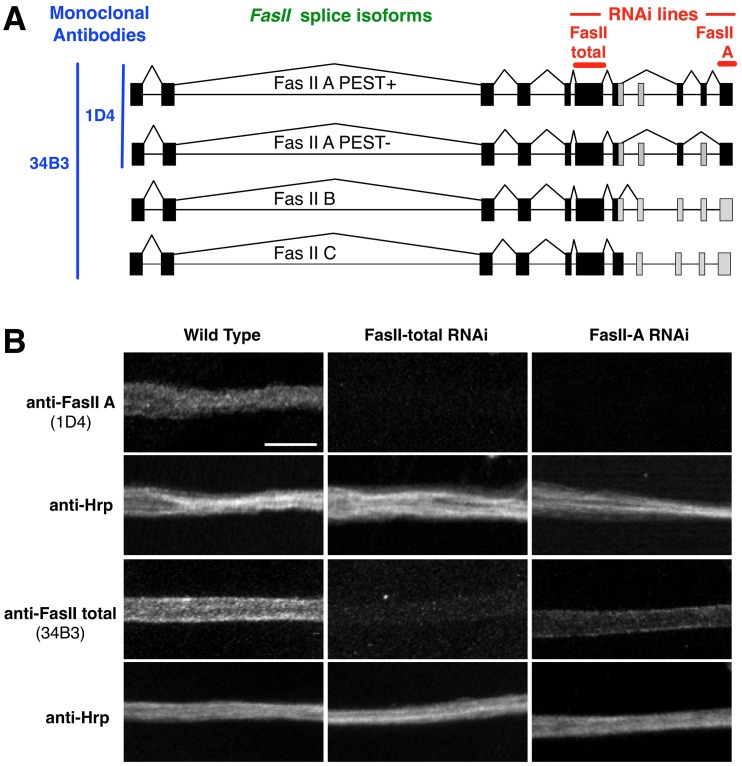
Inhibition of specific FasII isoforms using pBID-UAS-GGi. **A.** The *Fasciclin II* (*FasII*) gene has four splice isoforms, A PEST+, A PEST-, B, and C, that all include the first seven exons but differ in the inclusion (black) or exclusion (grey) of exons at the 3′ end of the gene. The monoclonal anti-FasII antibody 34B3 recognizes an epitope in the extracellular domain of all four isoforms (anti-FasII total). The monoclonal antibody 1D4 recognizes an epitope in the intracellular domain of both FasII-A-PEST+ and FasII-A-PEST- (anti-FasII A). An existing UAS RNAi line (FasII-total RNAi) targets an exon common to all four isoforms. FasII-A RNAi, generated using pBID-UAS-GGi, targets an exon found in only the A isoforms. **B.** Wild type motor neuron axons are labeled by anti-FasII A and anti-FasII total. Neuronal membranes are labeled by anti-Hrp. When FasII expression is inhibited by expression of FasII-total RNAi with Da-Gal4, FasII cannot be detected in axons by either antibody. In contrast, inhibition of FasII-A isoforms alone by expression of FasII-A RNAi with Da-Gal4, eliminates labeling by anti-FasII A but FasII protein is still detectible in axons with anti-FasII total. This indicates that protein isoforms other than FasII A isoforms are present in motor axons. Scale Bar  = 10 µm.

To test the functionality of this vector, we inhibited alternative splice isoforms of Fasciclin II (FasII), the *Drosophila* ortholog of Neural Cell Adhesion Molecule (NCAM) [37]. The *fasII* gene generates at least four protein isoforms by alternative splicing of several 3′ exons [38], [39]. Identical exons are employed by all four FasII transcripts to generate the extracellular domain of the protein and this region is targeted by an existing transgenic UAS RNAi construct that targets FasII (UAS FasII-total RNAi) [18] (Figure 5A). Alternative splicing of 3′ *fasII* exons produce one of either of two FasII-A transmembrane isoforms (designated PEST+ or PEST-), a putative GPI linked isoform (FasII-C) and a fourth isoform (FasII-B) that remains poorly characterized. All isoforms of FasII protein are detectible with the monoclonal antibody 34B3 while only FasII-A isoforms are detected by the monoclonal antibody 1D4 [40]–[42] (Figure 5A). Using either of these antibodies, FasII protein is detectible in motor neuron axons (Figure 5B), indicating that FasII-A isoforms are found in axons, however it was unknown if other isoforms were also localized there.

To determine which FasII protein isoforms are present in motor axons, we targeted expression of FasII-A isoforms alone by cloning a sequence corresponding to *fasII* exon 12, common to both FasII-A isoforms but not FasII-B or FasII-C, into pBID-UAS-GGi to generate UAS FasII-A RNAi (Figure 5A) [43]. We then compared the effect on FasII expression by RNAi inhibition with this construct compared to inhibition by UAS FasII-total RNAi when driven ubiquitously by Da-Gal4. When we inhibited FasII expression with UAS FasII-total RNAi, we could not detect FasII protein in motor axons with either the 34B3 or 1D4 antibodies, indicating potent inhibition of all FasII protein expression (Figure 5B). When we targeted only the FasII-A isoforms with UAS FasII-A RNAi, we also could detect no protein with the FasII-A specific 1D4 antibody in motor axons consistent with effective targeting of these isoforms. However, when we stained these axons with 34B3 antibody could still detect some FasII protein expression indicating that either or both of the FasII-B or FasII-C isoforms are also found in motor neuron axons (Figure 5B). Therefore, our results show that the pBID-UAS-GGi vector facilitates the generation of custom UAS RNAi constructs, for example to target distinct mRNA splice isoforms, that can serve as a useful complement and extension to existing whole genome RNAi libraries for the analysis of *Drosophila* genes.

## Discussion

Analysis of transgenic genes and proteins in *Drosophila* has benefited from increasingly sophisticated tools and methods [7]. A recent advance is the ability to reproducibly target transgenes to genomic loci using ϕC31 phage integrase based DNA recombination [4]. Following this initial description, ϕC31 transgenic technology has been refined and improved by the construction of stable transgenic integrase sources and high expression landing sites [6], [9], [44]. Innovative applications of ϕC31 recombination have been developed such as the iterative modification of genomic loci (reviewed in [45]) or transposon based alteration of endogenous genes [46] and large scale libraries of RNAi transgenes have been generated using the system [34]. Here we describe a modular vector toolset designed to facilitate gene analysis by allowing equivalent expression of transgenes inserted using ϕC31 recombination at different *attP* landing sites, to expedite labeling of transgenes with fluorescent proteins or epitope tags and to allow inhibition of protein isoform expression by RNAi. Modular cloning tools have previously been developed for *Drosophila* P-elements (unpublished, http://emb.carnegiescience.edu/labs/murphy/Gateway%20vectors.html) and for *Caenorhabditis elegans,* where a modular restriction enzyme based vector series [47] has been developed upon to encompass a large variety of applications.

Our vectors offer a number of advantages but also some limitations over existing reagents. First, by design, our vectors incorporate insulator sequences to allow equivalent transgene expression independent of the *attP* landing site. For example, reporter constructs lacking insulators exhibit large differences in expression levels from *attP18*, *attP40* and *attP2* landing sites [9], [10] in contrast to our insulated constructs where expression was similar. Homogenous expression from different landing sites is advantageous for many experimental scenarios, for example the comparison of e.g. wild-type and mutant transgenes [22]. However, in other contexts this may be a disadvantage, if for example a series of transgenes with different expression levels is desired. It may be possible to achieve incremental expression levels of transgenes from these vectors by modifying the number of UAS binding sites which has been shown to change expression level in other constructs [10], [18], [19]. Furthermore, while we found equivalent expression from three commonly used *attP* landing sites, we cannot exclude the possibility that expression at other landing sites may not be similar due to incomplete protection by the insulating elements. Also for transgenic animal identification, our constructs use the mini-*white* gene, a common and easy to use selectable marker [48]–[50]. However for some purposes, such as courtship assays, ectopic *white* containing transgenic constructs can cause aberrant behavior [51], [52] and other markers may be preferred. Alternatively, integration of our vectors into *ZH-attP* landing sites [6] will cause the mini-*white* gene to be flanked by both a *loxP* site derived from the pBID vector and *loxP* site present in these landing sites. Transgenic Cre can then be used delete the region between the *loxP* sites removing the mini-*white* selectable marker and other plasmid sequences while leaving the remainder of transgenic construct intact [6].

Second, for all UAS vectors apart from pBID-UAS-GGi, we used a *Drosophila* Synthetic Core Promoter as the basal promoter [17]. As we have shown, this has the major advantage of avoiding non-specific ‘leaky’ transgene expression, a problem which has limited the usefulness of other insulated vectors [9], [14]. The transgenic UASC TeTxLC lines we generated with these constructs, for example, do not perturb circadian behavior in the absence of Gal4 unlike existing P-element based constructs (Michael Nitabach and Justin Blau, *pers.comm*) consistent with little or no Gal4 independent expression. However, as described in other studies [10], we also find that this promoter produces less Gal4 induced transgene expression than vectors with *hsp70* based basal promoters. This is a particular disadvantage for transgenic RNAi constructs where high levels of hairpin production are desirable [18], [53]. Indeed, we initially constructed a pBID-UASC-GGi vector that employed the DSCP promoter, however transgenes generated with this construct produced only low to moderate inhibition of gene expression (data not shown) and we therefore built the hsp70 based pBID-UAS-GGi vector which proved more effective. Again it is possible that the addition of more UAS binding sites [10] or perhaps translational enhancers [54] might alleviate this disadvantage.

Third, the majority of our vectors use Gateway cloning to introduce transgenes. This *in vitro* recombination based system allows robust high throughput parallel cloning of inserts into multiple vectors, easier cloning of large or complex inserts that may be refractory to restriction enzyme based cloning and simplifies the generation of fusion proteins [16], [55], [56]. In addition, Gateway compatible vectors are readily available for a number of biological applications such as *Drosophila* S2 or mammalian cell expression that can aid in protein analysis and compatible cDNA libraries exist for many mammalian genes. It is also noteworthy that as part of our construction process, we generated several intermediate vectors (pMartini-Gateway series) that can facilitate the subcloning of Gateway cassettes and the generation of new ‘destination’ vectors (see materials and methods). However, for ‘one off’ construct generation Gateway technology may be more cumbersome and expensive due to the necessity to generate an ‘entry’ clone prior to recombination into the final ‘destination’ vector such as the pBID ‘G’ vectors. However, it is possible to avoid the entry vector step and recombine PCR products directly into destination vectors [57] which may mitigate this disadvantage.

Finally, we have generated constructs that allow fusion to the yellow fluorescent protein mVenus [27], the orange/red fluorescent protein TagRFP-T [29], [30] and the Flag epitope [32]. Both mVenus and TagRFP-T are monomeric, produced bright fluorescence in our hands and fusion of these proteins to several transgenes did not disrupt protein function assayed by transgenic mutant rescue (e.g. alternative splicing factors [43]). Interestingly however, fusion of mVenus to the C-terminus of TeTxLC partially inhibited the ability of the toxin to block neurotransmitter release in *Drosophila* (data not shown), even though it was expressed at similar levels to untagged constructs and similar fusions of TeTxLC to fluorescent proteins have been employed in hippocampal neurons and transgenic zebrafish [58], [59]. Therefore the usefulness of this fusion protein is limited. In addition to fluorescence, mVenus can be also be readily detected by immunohistochemistry or western blotting with specific antibodies (most anti-GFP antibodies also detect mVenus), similar to Flag tagged transgenes. While antibodies against TagRFP-T are also available and work in *Drosophila* tissues [60], we added a Myc epitope to the TagRFP-T constructs [31] to allow more flexibility in antibody species choice. Development of novel and improved fluorescent proteins is rapid [61] and we envision the generation of fusion constructs with additional colors or other useful properties in future studies. We also hope that the *Drosophila* community will take advantage of the modularity of our constructs to expand upon and improve these vectors with further innovations.

## Materials and Methods

### Molecular Biology – Vector Assembly

All vector constructs have been deposited at Addgene http://www.addgene.org/a non-profit plasmid repository.

#### Construction of pBID

A three-fragment ligation was used to generate the pBID vector. A 473-bp DNA fragment containing the Gypsy insulator sequence [21] was PCR amplified from wild type Canton S genomic DNA with BamHI-Gypsy and Gypsy-MCS primers and cloned into pCR8GW-TOPO vector (Invitrogen), resulting in pCR8GW-BamHI-Gypsy-MCS. A 481-bp DNA fragment gypsy insulator sequence was PCR amplified from *Drosophila* wild type Canton S genomic DNA with MCS-Gypsy and Gypsy-BamHI primers and cloned in pCR8GW-TOPO vector, resulting in pCR8GW-MCS-Gypsy-BamHI. The pUASTattB [6] (gift from Konrad Basler, University of Zurich) was digested with BamHI to generate a fragment containing the mini-*white*, *loxP*, attB, *AmpR* and bacterial replication regions while removing other sequences. A 460-bp gypsy fragment released from pCR8GW-BamHI-Gypsy-MCS with BamHI/NotI and a 468-bp gypsy insulator released from pCR8GW-MCS-Gypsy-BamHI with BamHI/NotI were ligated into BamHI fragment to generate the pBID vector.

#### Construction of pMartini Gate A R1-R2, pMartini Gate A R2-R1, pMartini Gate B R1-R2, pMartini Gate B R2-R1, pMartini Gate C R1-R2 and pMartini Gate C R2-R1

The 1711-bp Gateway Cassette Reading Frame A (Invitrogen) was blunt end cloned into the EcoRV site of pMartini (gift from Nick Brown, Cambridge University), resulting in pMartini Gate A R1-R2 and in the opposite orientation pMartini Gate A R2-R1. The 1713-bp Gateway Cassette Reading Frame B (Invitrogen) was blunt end cloned into the EcoRV site of pMartini, resulting in pMartini Gate B R1-R2 and in the opposite orientation pMartini Gate B R2-R1. The 1714 bp Gateway Cassette Reading Frame C.1 (Invitrogen) was blunt end cloned into the EcoRV site of pMartini, resulting in pMartini Gate C R1-R2 and in the opposite orientation pMartini Gate C R2-R1.

#### Construction of pBID-G

A 1763-bp Gateway cassette was excised from pMartini Gate A R2-R1 with XhoI/XbaI and cloned into XhoI/XbaI sites of pBID vector to generate pBID-G.

#### Construction of pBID-UAS

The 10X UAS region of pGD264 (gift from Barry Dickson, IMP Vienna) was excised with NotI and modified with Klenow, subsequently digested with BglII and the 526-bp fragment was gel purified. pUASTattB was treated sequentially with HindIII, Klenow and BglII to remove UAS sequences**.** The 526-bp containing the 10X UAS region was then ligated into this vector. The resulting p10xUASTattB was digested with BamHI, and a 1264-bp BamHI fragment was inserted into BglII site of pBID to generate pBID-UAS.

#### Construction of pBID-UASC

pBID-UASC was constructed by three-fragment ligation. To this end, 255-bp 5xUAS sequence was PCR amplified from pUAST [2] with Pry1 and SacI-UAS reverse primers and cloned into pCR8GW-TOPO (Invitrogen) to create pCR8GW-5xUAS. Meanwhile, a 344-bp DSCP (*Drosophila* Synthetic Core Promoter) sequence was PCR amplified from pBPGUw (gift from Gerald Rubin, Janelia Farm) with Gateway attR2 and EcoRI-DSCP reverse primers, and a 151-bp fragment released from the 344-bp DSCP fragment with SacI/EcoRI was cloned into SacI/EcoRI sites of pBluescript SK(+), resulting in pBS-DSCP. A 132-bp HindIII/SacI 5xUAS fragment of pCR8GW-5xUAS and a 159-bp SacI/EcoRI DSCP fragment of pBS-DSCP were inserted in HindIII/EcoRI sites of pBID-UAS, giving rise to pBID-UASC.

#### Construction of pBID-UASC-G

A 1763-bp Gateway cassette was released from pMartini Gate A R2-R1 with XhoI/XbaI and cloned into XhoI/XbaI sites of pBID-UASC, giving rise to pBID-UASC-G.

#### Construction of pBID-UASC-VG

To construct a mVenus-Gateway cassette fusion, a 739-bp mVenus fragment with a *Drosophila* Kozak sequence added prior to start codon was PCR amplified from pSK2691 (gift from Gary Struhl, Columbia University) with HindIII-BglII-mVenus and mVenus-no stop-SphI primers, digested with HindIII/SphI and cloned into HindIII/SphI sites of pMartini Gate A R1-R2. The resulting pMartini-mVenus-G was digested with BglII/XhoI and the released 2480-bp fragment was inserted into BglII/XhoI sites of pBID-UASC, giving rise to pBID-UASC-VG.

#### Construction of pBID-UASC-GV

To construct a Gateway-mVenus fusion, a 719-bp mVenus fragment was PCR amplified from pSK2691 (gift from Gary Struhl, Columbia University) with SphI-mVenus and mVenus-stop-XbaI primers, digested with SphI/XbaI and cloned into SphI/XbaI sites of pMartini Gate B R2-R1. The resulting pMartini-G-mVenus was digested with XhoI/XbaI and the released 2476-bp fragment was inserted into XhoI/XbaI sites of pBID-UASC, giving rise to pBID-UASC-GV.

#### Construction of pBID-UASC-MRG

119-bp 3xMyc tag with a *Drosophila* Kozak sequence added prior to the start codon was PCR amplified from pCMV-3Tag-4C (Stratagene) with fly HindIII-3xMyc FWD and 3xMyc-XbaI REV primers. TagRFP-T was PCR amplified as 749-bp XbaI-TagRFP2-SphI fragment from pSK3007 (gift from Gary Struhl, Columbia University) with XbaI-TagRFP2 and TagRFP2-no stop-SphI primers. The 3x Myc fragment was cut with HindIII/XbaI, the TagRFP-T fragment with XbaI/SphI, and both were simultaneously ligated into pMartini Gate A R1-R2 prepared with HindIII/SphI. The resulting pMartini-d3myc-TagRFP2-G was cut with XhoI and released a 2626-bp d3myc-TagRFP2-G fragment, inserted into the XhoI site of pBID-UASC, giving rise to pBID-UASC-MRG.

#### Construction of pBID-UASC-GRM

A 113-bp 3x Myc tag was PCR amplified from pCMV-3Tag-4C (Stratagene) with XbaI-3xMyc FWD and 3xMyc-stop-HindIII REV primers. TagRFP-T was PCR amplified as 749-bp SphI-TagRFP2-XbaI fragment from pSK3007 (gift from Gary Struhl, Columbia University) with SphI-TagRFP2, TagRFP2-no stop-XbaI primers. The 99-bp 3x Myc fragment was cut with XbaI/HindIII, the 731-bp TagRFP-T fragment with SphI/XbaI, and both were simultaneously ligated into pMartini Gate B R2-R1 prepared with SphI/HindIII, creating pMartini-G-TagRFP2–3Myc. A 2606-bp G-TagRFP2–3Myc EcoRI/XhoI fragment derived from pMartini-G-TagRFP2–3Myc was cloned into EcoRI/XhoI site of pBID-UASC, giving rise to pBID-UASC-GRM.

#### Construction of pBID-UASC-FG

A 3xFLAG tag followed by Gateway cassette was excised from pAFW (Terence Murphy, Carnegie Institute, *Drosophila* Genomics Resource Center (DGRC)) with XhoI/NheI and cloned into XhoI/XbaI sites of pBID-UASC, resulting in pBID-UASC-FG.

#### Construction of pBID-UAS-GGi

The pRISEftz plasmid [36](gift from Takefumi Kondo, Nara Institute) was digested partially with XbaI and completely with BglII. The released 3613-bp fragment containing two Gateway cassettes in opposite orientations separated by a ftz intron was inserted into BglII/XbaI sites of pBID-UAS, resulting in pBID-UAS-GGi.

### Molecular Biology – Validation Transgene Assembly

#### General methods and insert confirmation

PCR was performed using AccuPrime *Pfx* SuperMix (Invitrogen). White and attB primers were used for sequencing the insertion in pBID vectors. The DSCP primer was used for sequencing inserts after a DSCP. The SV40 pA primer was used for sequencing inserts in front of SV40 polyA. All Primers are described in Table S1.

#### Construction of pBID-UASC-dsEGFP

To generate pBID-UASC-dsEGFP, dsEGFP was PCR-amplified from pd2EGFP-N1 (Clontech) using dsEGFP F and R primers. By PCR, an NLS sequence was added the N-terminus and NotI site inserted between amino acids 154 and 155 to facilitate future reporters. The construct was inserted into pBID-UASC using BglII and XhoI.

#### Construction of pBID-UASC-TeTxLC

Tetanus toxin light chain (TeTxLC) construct pBID-UASC-TeTxLC was derived by an LR reaction between pBID-UASC-G and entry plasmid pCR8GW-TeTxLC (gift from Ben Jiwon Choi and Joseph Gogos, Columbia University).

#### Construction of pBID-UASC-TeTxLC-mVenus

The TeTxLC with a C-terminal mVenus fusion gene construct pBID-UASC-TeTxLC-mVenus was derived by an LR reaction between pBID-UASC-GV and the entry plasmid pCR8GW-TeTxLC-no-stop (gift of Ben Jiwon Choi, Columbia University).

#### Construction of pBID-UASC-mVenus-TBPH

*Drosophila* TBPH [62] with an N-terminal fusion to mVenus was generated by an LR reaction between pBID-UASC-VG and the TBPH entry plasmid pCR8GW-TBPH [22].

#### Construction of pBID-UASC-3myc-tRFP-Caz

*Drosophila* Caz with an N-terminal MyctRFP fusion was generated by an LR reaction between pBID-UASC-MRG and the Caz entry plasmid pCR8GW-Caz [22].

#### Construction of pBID-UASC-Flag-Caz

*Drosophila* Caz with an N-terminal Flag fusion was derived by an LR reaction between pBID-UASC-FG and the Caz entry plasmid pCR8GW-Caz [22].

#### Construction of pBID-UAS-FasII-A RNAi

UAS-FasIIRNAi-A [43] was generated by PCR amplification of the sequence corresponding to FasII exon 12 from total cDNA derived from larval brains using primers FasII-RNAi-A F and R**.** The PCR product was TOPO cloned into the pCR8GW-TOPO vector (Invitrogen) to generate pCR8GW-FasII-A. An LR reaction between pCR8GW-FasII-A and pBID-UAS-GGi was used to generate pBID-UAS-FasII-A RNAi.

### Drosophila Genetics

#### Gal4 lines

The GAL4 lines used were the eye driver GMR-GAL4 [63], the motor neuron driver OK6-GAL4 and the muscle driver G14-GAL4 [28] and the ubiquitous driver Da-Gal4 [64].

#### Transgene generation

Transgenes were inserted into the *attP18* (X chromosome), *attP40* (chromosome II), or *attP2* (chromosome III) landing sites [9] using established methods (Genetic Services, Cambridge.).

### Immunohistochemistry

Dissection and immunohistochemistry was performed as previously described [65], [66]. DAPI (Sigma) was used at 1∶2,000 dilution for 10 min at room temperature. Antibodies used were: Chicken anti-GFP (1∶2,000, Abcam), mouse anti-bruchpilot (nc82, 1∶500, DSHB), mouse anti-Myc tag (9E10, 1∶100, DSHB), mouse anti-FLAG (M2, 1∶1,000, Sigma), goat anti-mouse IgG conjugated to Cy3 (1∶1,000, Molecular Probe), goat anti-mouse IgG conjugated to Alex488 (1∶2,000, Molecular Probe), mouse anti-FasII TM (1D4, 1∶900, DSHB from C. Goodman), mouse anti-FasII total (34B3, 1∶20, DSHB from C. Goodman).

### Western Blotting

Adult fly heads were directly homogenized in sample loading buffer. The heads extract was centrifuged at 16,000 rpm for 5 minutes, subjected to 10% or 12% SDS-PAGE, transferred to Protran® nitrocellulose membrane (Whatman), and detected with Pierce® ECL Western Blotting Substrate (Thermo Scientific). The primary antibodies used were: mouse anti-tetanus toxin light chain (1∶200, gift from Heiner Niemann), rabbit anti-GFP (1∶2,000, Invitrogen), mouse anti-β-tubulin (E7, 1∶1,000, DSHB).

## Supporting Information

Table S1 **Sequence of PCR primers employed.**
(PDF)
