# Supplementary material for: A Modular Toolset for Recombination Transgenesis and Neurogenetic Analysis of Drosophila
Source: PLoS One. 2012 Jul 25;7(7):e42102. doi: 10.1371/journal.pone.0042102 (PMC3405054; doi:10.1371/journal.pone.0042102)
Supplement: Table S1 — Sequence of PCR primers employed. (PDF) [file pone.0042102.s001.pdf]

Table S1 Primer Sequences

Primer	Sequence (5' → 3')
BamHI-Gypsy	AATGGATCCTGGCCACGTAATAAGTGTGCGTTG
Gypsy-MCS	AATGCGGCCGCTTAATTAAGCGAATTCAGATCTGTTGTTGGTTGGCACACCACA
MCS-Gypsy	AATGCGGCCGCGGCTCGAGAAGGTACCGGTCTAGAAGATCTTGGCCACGTAATAAGTGTGC
Gypsy-BamHI	AATGGATCCGTTGTTGGTTGGCACACCACA
Pry1	CCTTAGCATGTCCGTGGGGTTTGAAT
SacI-UAS reverse	AAAAGAGCTCGCTAGAGTCTCCGCTCG
Gateway attR2	TGTCAGGCTCCCTTATACAC
EcoRI-DSCP reverse	TATTGAATTCGTTTGGTATGCGTCTTGT
HindIII-BglII-mVenus	AAAAAAGCTTAGATCTATCAACATGGTGAGCAAGGGCGAGGAGC
mVenus-no stop-SphI	AAAAGCATGCCTTGTACAGCTCGTCCATGCCG
SphI-mVenus	AAAAGCATGCGTGAGCAAGGGCGAGGAGC
mVenus-stop-XbaI	AAAATCTAGATTACTTGTACAGCTCGTCCATGCCG
fly HindIII-3xMyc FWD	AAAAAAGCTTATCAACATGGAGCAGAAACTCATC
3xMyc-XbaI REV	AAAATCTAGACAGATCTTCCTCAGA
XbaI-TagRFP2	AATTTCTAGAGTGAGCAAGGGCGAGGAGCTGATTAAG
TagRFP2-no stop-SphI	AAAAGCATGCCTTGTACAGCTCGTCCATGCCATTAAGTTT
XbaI-3xMyc FWD	TAAATCTAGAGAGCAGAAACTCATC
3xMyc-stop-HindIII REV	TTTAAAGCTTCTACAGATCTTCCTCAGA
SphI-TagRFP2	AAAAGCATGCGTGAGCAAGGGCGAGGAGCTGATTAAG
TagRFP2-no stop-XbaI	AAAATCTAGACTTGTACAGCTCGTCCATGCCATTAAGTTT
dsEGFP F	TTCTAGAATGGTGAGCAAGGGCGAGGAG
dsEGFP R	AAGGTACCTTAGTCCATCCCGCTCTCCTGG
FasII-RNAi-A F	CACCCCAGCTGGTAAAGGAGCCACC
FasII-RNAi-A R	GGCGTGCTCATGGGCGAACCG
white primer	TGGTGGGCATAATAGTGTTGTT
attB primer	GGATCAACTACCGCCACCT
DSCP primer	CGTGCCGCTGCCTTCGTT
SV40 pA primer	CCTTAGAGCTTTAAATCTCTGTAGG
